# Rectal Lymphogranuloma Venereum, France

**DOI:** 10.3201/eid1103.040621

**Published:** 2005-03

**Authors:** Magid Herida, Patrice Sednaoui, Elisabeth Couturier, Didier Neau, Maïthe Clerc, Catherine Scieux, Gerard Kreplak, Véronique Goulet, Françoise F Hamers, Bertille de Barbeyrac

**Affiliations:** * Institut de Veille Sanitaire, Saint-Maurice, France;; † Institut Alfred Fournier, Paris, France;; ‡ Hospital Pellegrin, Bordeaux, France;; § Université Bordeaux 2, Bordeaux, France;; ¶ Hospital Saint-Louis, Paris, France;; # Laboratoire du Chemin Vert, Paris, France

**Keywords:** France, lymphogranuloma venereum, MSM, letter

**To the Editor:** Lymphogranuloma venereum (LGV), a sexually transmitted disease (STD) caused by *Chlamydia trachomatis* serovars L1, L2, or L3, is prevalent in tropical areas but occurs sporadically in the western world, where most cases are imported (*1*). LVG commonly causes inflammation and swelling of the inguinal lymph nodes, but it can also involve the rectum and cause acute proctitis, particularly among men who have sex with men. However, LGV serovars of *C. trachomatis* remain a rare cause of acute proctitis, which is most frequently caused by *Neisseria gonorrhoeae* or by non-LGV *C. trachomatis* (*2*). In 1981, in a group of 96 men who have sex with men with symptoms suggestive of proctitis in the United States, Quinn et al. found that 3 of 14 *C. trachomatis* infections were caused by LGV serovar L2 (*3*). In France, 2 cases of rectal LGV were reported in an STD clinic in Paris from 1981 to 1986 (*4*). In 2003, an outbreak of 15 rectal LGV cases was reported among men who have sex with men in Rotterdam; 13 were HIV-infected, and all reported unprotected sex in neighboring countries, including Belgium, France, and the United Kingdom (*5*). At the same time, a rise in *C. trachomatis* proctitis (diagnosed by using polymerase chain reaction [PCR]; [Cobas Amplicor Roche Diagnostic System, Meylan, France]) was detected in 3 laboratories in Paris and in the *C. trachomatis* national reference center located in Bordeaux. To identify the serovars of these *C. trachomatis* spp., all stored rectal specimens were analyzed by using a nested *omp*1 PCR-restriction fragment length polymorphism assay. The amplified DNA product was digested by restriction enzymes. Analysis of digested DNA was performed by electrophoresis. Patterns were compared visually with reference patterns (*6*).

From January 1, 2003, to March 31, 2004, a total of 44 of 124 male rectal swabs were positive for *C. trachomatis*. Of those, 38 were identified as belonging to the L2 serotype, which confirms the diagnosis of rectal LGV. Epidemiologic information was retrospectively obtained by clinicians through review of medical records, telephone interview, or both. A complete history was available for 14 of the 38 cases. All 14 men reported unprotected anal sex with anonymous male sex partners in France, and none reported a stay in an LGV-endemic area. Their mean age was 40 years (31–50); 8 were HIV-infected, and 9 had another concomitant STD. The mean duration of symptoms before LGV diagnosis was 50 days (range 11–120 days). All 14 patients had symptoms of acute proctitis, including rectal pain, discharge, and tenesmus, and 3 (all HIV-infected) had fever. Deep, extended rectal ulcerations were reported in 8 patients, 3 of whom were HIV-infected and had lesions suggestive of rectal carcinoma. In 1 patient in whom a late diagnosis was made 4 months after the onset of symptoms, a rectal tumorlike stricture was observed. All 14 patients were treated with tetracycline for a mean duration of 16 days (range 10–60 days).

An information campaign among microbiologists and clinicians and a sentinel LGV surveillance system were launched in April 2004. Subsequently, LGV was diagnosed in 65 additional male patients, some retrospectively. In total, rectal LGV was diagnosed in 103 patients from July 2002 to August 2004 (Figure).

**Figure:**
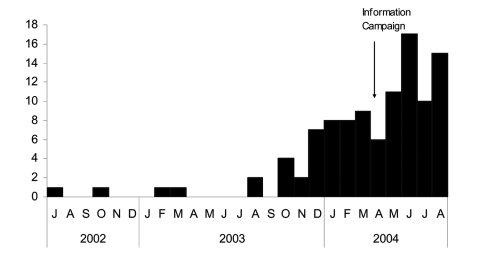
Number of rectal lymphogranuloma venereum cases diagnosed in men in France, July 2002–August 2004.

Prompt diagnosis and treatment is indeed paramount to prevention and control. Diagnosis may be further hampered because rectal LGV may mimic other conditions such as rectal carcinoma or Crohn disease. Treatment duration should be no shorter than 21 days, and follow-up examinations should be conducted until all signs and symptoms have resolved (*7**,**8*). If left untreated, rectal LGV could lead to serious complications such as rectal stricture (*1*). If recently exposed to infection, sexual contacts should receive prophylactic treatment to prevent reinfection and to eliminate a potential reservoir. The emergence of rectal LGV, characterized by deep mucosal ulcerations and frequently occurring in HIV-infected men who have sex with men, is a serious concern for the gay community in Europe.
